# Millennial-scale faunal record reveals differential resilience of European large mammals to human impacts across the Holocene

**DOI:** 10.1098/rspb.2015.2152

**Published:** 2016-03-30

**Authors:** Jennifer J. Crees, Chris Carbone, Robert S. Sommer, Norbert Benecke, Samuel T. Turvey

**Affiliations:** 1 Institute of Zoology, Zoological Society of London, Regent's Park, London NW1 4RY, UK; 2 Department of Landscape Ecology, Institute for Natural Resource Conservation, University of Kiel, Olshausenstrasse 75, 24118 Kiel, Germany; 3 Department of Natural Sciences, German Archaeological Institute, Im Dol 2-6, Berlin 14195, Germany

**Keywords:** environmental baselines, extinction filter, Late Quaternary, megafaunal extinction, range shifts, zooarchaeology

## Abstract

The use of short-term indicators for understanding patterns and processes of biodiversity loss can mask longer-term faunal responses to human pressures. We use an extensive database of approximately 18 700 mammalian zooarchaeological records for the last 11 700 years across Europe to reconstruct spatio-temporal dynamics of Holocene range change for 15 large-bodied mammal species. European mammals experienced protracted, non-congruent range losses, with significant declines starting in some species approximately 3000 years ago and continuing to the present, and with the timing, duration and magnitude of declines varying individually between species. Some European mammals became globally extinct during the Holocene, whereas others experienced limited or no significant range change. These findings demonstrate the relatively early onset of prehistoric human impacts on postglacial biodiversity, and mirror species-specific patterns of mammalian extinction during the Late Pleistocene. Herbivores experienced significantly greater declines than carnivores, revealing an important historical extinction filter that informs our understanding of relative resilience and vulnerability to human pressures for different taxa. We highlight the importance of large-scale, long-term datasets for understanding complex protracted extinction processes, although the dynamic pattern of progressive faunal depletion of European mammal assemblages across the Holocene challenges easy identification of ‘static’ past baselines to inform current-day environmental management and restoration.

## 1. Introduction

Extinction constitutes a process rather than a single event, with the final disappearance of the last individual of a species merely the endpoint of an often protracted series of regional population losses which may take decades, centuries or even longer to run their course [1]. A principal aim of conservation biology is therefore to develop methods to characterize this process, and greater emphasis is now placed on understanding the spatio-temporal and ecological dynamics of localized extirpations, population declines and range collapses, in order to identify general patterns of decline and provide predictive power for conservation management of threatened species [2–5].

Humans are now a dominant driver of patterns in global biodiversity, and well-documented ongoing anthropogenic transformation of the biosphere is responsible for catastrophic recent declines across a broad range of taxa [6,7]. However, human activities have also substantially affected species diversity and ecosystem structure throughout the historical period and recent prehistory. Indeed, few (if any) of the nearly 800 documented mammal and bird species-level extinctions that have taken place during the relatively climatically stable Holocene epoch (11 700 years ago–present, the time interval since the end of the last Ice Age glaciation) can be interpreted as non-anthropogenically mediated [8]. There is therefore an increasing awareness of the need to integrate long-term datasets into conservation research and environmental management, to provide novel insights into population trends, extinction dynamics, and the status of both species and ecosystems that are not available from short-term ecological studies [9–12].

However, the use of long-term data in ecology and conservation remains limited, owing to the lack of standardized scientific monitoring data beyond the recent past. Current indicators for measuring population declines rarely use baselines older than AD 1500 [13], and ecological monitoring data used to measure trends in biodiversity typically span only a few decades [14–16]. Likewise, large amounts of data across wide spatial and temporal scales are required to characterize changes in species' geographical ranges across the entire duration of population declines, but studies of range dynamics over time are typically limited to comparisons between present-day species distributions and single ‘historical’ maps, usually representing geographical range estimates no more than a few hundred years old [2–4,17,18].

Consideration of ecological time-series data from the recent past alone may be supported by the increasing recognition of a modern ‘Anthropocene’ epoch, defined by qualitatively more intensive human pressures on global ecosystems during the past few decades or centuries [19]. However, using a recent baseline imposes an explicit ‘extinction filter’ [20] that excludes particularly vulnerable populations and species that were lost due to older human impacts, which has major implications for our insights into extinction ecology [21]. Analyses based on such reduced subsets of surviving taxa can provide only an incomplete understanding of patterns of vulnerability and resilience shown by different species to human impacts through time. For example, regional mammal faunas from which the most susceptible species have become extinct now appear less threatened, and higher-order taxonomic groups containing elevated numbers of recently extinct species now show only average levels of current extinction risk [22]. Similarly, although small geographical range size has been proposed as a key predictor of extinction risk in mammals [23,24], this may actually represent a circular predictor if historical depletion has occurred in response to past human impacts [25–27].

Employing restricted time windows for ecological analysis could have particularly significant implications for understanding extinction dynamics and vulnerability in geographical regions with long histories of human occupation, notably continents such as Europe [28]. This region represents a unique study system across which to investigate long-term human impacts on biodiversity, as a wealth of dated occurrence records spanning the Holocene, comprising subfossil, zooarchaeological, historical and ecological data, are available for many European large mammal species. Recent investigation of the European Holocene zooarchaeological record has demonstrated that it is possible to use this long-term archive to reconstruct high-resolution extinction dynamics for specific large mammal taxa, revealing that some species experienced spatially complex patterns of staggered population extirpation across Europe before the recent historical era [29]. This archive also has the potential to enable assemblage-wide analyses of extinction, for example, to determine the duration, magnitude and selectivity of prehistoric human-caused continental extinction ‘events’. It can also be used to investigate whether co-occurring species or species groups showed either congruent responses to past pressures or marked individualistic differences in timing or magnitude of population losses. However, it is important to recognize that the zooarchaeological record, much like the wider fossil record, suffers from both incompleteness and bias, and does not represent a systematic sample of past species diversity across space or time [30]. This major concern therefore needs to be accounted for when analysing past faunal data, to avoid misinterpreting past patterns of population loss and extinction [31].

Faunal research for the Holocene to date has predominantly consisted of documenting global species-level extinction ‘events’ [8]. However, while we therefore have increasing information on taxonomic losses across the Holocene, we lack a robust understanding of the dynamics and ecology of these extinctions [32]. Studies have tended to focus on oceanic island faunas that have experienced elevated levels of species extinction associated with the arrival of humans and commensal mammal predators [8]. By contrast, there has been little research into continental mammal losses during the Holocene, partly due to the reduced number of global-level continental species extinctions after the Late Pleistocene and before the recent historical era (the so-called ‘Holocene underkill’ [33]). For example, only one representative of Europe's recent continental large mammal fauna, the aurochs (*Bos primigenius*), has become globally extinct. Today this fauna includes many species that are of conservation concern and the focus of intensive management efforts [34,35], with particular attention paid to conservation of surviving European populations of large carnivores, considered to be a particularly vulnerable ecological guild [36–38]. While there have been several well-documented local extirpations of geographically discrete insular European mammal populations (e.g. in the British Isles [39,40]), the majority of previous assessments of Holocene extinctions in this region have been conducted at coarse species-level resolutions. Comparative patterns of population change across Europe's large mammal fauna during this interval, and even any evidence of human impact on these populations before the recent historical era, therefore remain largely unknown.

In the absence of a rigorous and standardized assessment of the Holocene record, it is not possible to determine whether current-day continent-wide disruption of large mammal faunal assemblages [18] represents a recent and rapid phenomenon or the culmination of a long-term process of progressive population attrition. Furthermore, we are unable to assess whether anthropogenic processes have affected different mammal species in a qualitatively and quantitatively similar way through time. As all Holocene mammal losses in Europe can uncontroversially be attributed to human activity [8,41], establishing a strengthened framework for understanding the spatio-temporal pattern of these losses across an entire fauna is essential in order to determine the duration, magnitude and selectivity of anthropogenic impacts on biodiversity, and thereby inform effective current-day management of threatened large mammals. We therefore used the extensive data on past distributions of large mammal species available in the Holocene zooarchaeological record as a proxy for ecological monitoring data to reconstruct millennial-scale patterns of mammalian extinction across Europe, within a robust quantitative framework that controlled for bias inherent in such a dataset.

## 2. Material and methods

### (a) Data collection

**Figure 1.**
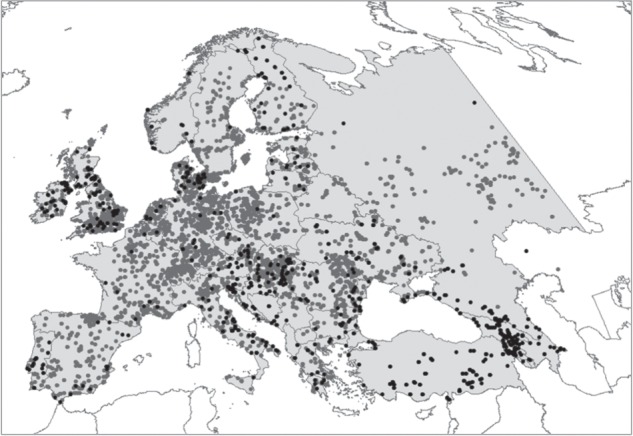
Map of Europe (10 417 608 km^2^ study area shown in grey), showing distribution of 18 670 zooarchaeological localities containing records of native mammal species. Data from original Holocene zooarchaeological database [41] shown in grey; additional data collected for this study shown in black.

An extensive database of 18 670 mammal zooarchaeological records spanning the Holocene of Europe [41], comprising a 10 417 608 km^2^ study area including Turkey and the Caucasus but excluding Iceland and the insular Mediterranean other than Sicily (figure 1; electronic supplementary material, table S1), was used as the basis for analysis. Additional data collection was undertaken to increase geographical sampling consistency, include more detailed coverage of previously undersampled regions (e.g. the Balkans, Turkey, the Caucasus) and include records published up to 2013 [42]. All records are associated with details of species, location (country, site, region, latitude, longitude) and date (absolute/relative) (electronic supplementary material, text S1). Species taxonomy follows Wilson & Reeder [43].

### (b) Data analysis

Methods for reconstructing ranges have been widely discussed [44,45], but with no common consensus on an absolute method that is appropriate across all types of data or analysis. Differences exist in the spatial patterning and quantity of data available for different species due to both pre- and post-excavation biases; variation in zooarchaeological species distribution records reflects complex variation in factors such as past settlement patterns and faunal exploitation by prehistoric communities, and also more recent archaeological search effort, in addition to underlying ecological variation in species distributions [46]. We therefore sought to avoid methods such as area of occupancy that rely heavily on the actual number and distribution of individual data points [13,45]. Such methods can also make assumptions about species ecology and habitat use when reconstructing past ranges, which may be confounded by uncertainty over whether distribution records represent optimal habitat or marginal refugia [47,48]. By contrast, while range extent can overestimate area of occupancy, it allows for reasonable comparison of relative changes in distribution between species and time periods for the same geographical area despite underlying unevenness in distribution of data points, especially when there is no evidence to suggest that some species distributions may be more subject to range-edge fluctuations than others. We therefore used the observed extent of occurrence, a measure of range extent calculated as the area within a convex hull polygon that encloses all the points with no internal angle measuring more than 180° [13]; this metric has previously been used to reconstruct species distributions using both Quaternary and older fossil data, which constitute presence-only data in contrast to many modern ecological datasets [49,50]. Species ranges were reconstructed and measured using the spatial mapping software ArcGIS v. 9.3 [51].

Data were analysed across seven well-established Holocene archaeological periods, which represent important shifts in human subsistence and/or technological change and which are broadly contemporaneous across Europe: Mesolithic (9500–5500 BC), Neolithic (5500–3000 BC), Bronze Age (3000–1000 BC), Iron Age (1000 BC–AD 0), Roman Age (AD 0–500), Early Medieval (AD 500–1000) and Late Medieval (AD 1000–1500). The recent historical era (AD 1500–present) was excluded from analysis, as there are few zooarchaeological records for this time period (faunal distributions are better represented by historical and modern ecological data), making direct comparison with older bone-based records difficult, and trends in species status are already assessed for this period [52].

We also checked for further possible spatial biases in our dataset arising from our measure of range extent, and found that number of zooarchaeological records and corresponding range size were positively correlated across all time periods and species (Pearson's *r* = 0.915, d.f. = 38, *p* < 0.05; electronic supplementary material, figure S1), probably reflecting the well-described positive abundance–occupancy relationship in ecology [53]. However, as a result of spatial, temporal and taxonomic variation in archaeological sampling [46], it was difficult to separate the relative influence of number of records versus genuine change in species range in driving observed variation in range size estimates through time. In order to deal with the potentially confounding influence of sample size, we used bootstrapping to establish null models of range size expectations for each archaeological period based on given sample sizes. For each species, all records across its Holocene range were randomly resampled 1000 times, with sample size constituting the number of records for any one time period, and range size was calculated for each run. This approach provided a measure of potential sampling variability of range size and extent based on the number of points available for each species and time period combination. Upper and lower 95% confidence intervals and mean range from the 1000 runs were then calculated and plotted together with the observed range extent. If the observed range fell outside these confidence intervals, this was interpreted as representing a genuine, statistically significant deviation from the expected range size for the species. We also combined the data into discrete categories for body mass (above and below 100 kg) and trophic level (herbivore/carnivore) to test for differences in patterns of range decline across these ecological groupings.

## 3. Results

**Figure 2.**
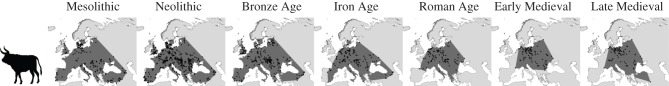
Geographical range estimates for aurochs from the Mesolithic to the Late Medieval, with range extent based on distribution of zooarchaeological records in each time period (black points).

Overall spatial coverage of zooarchaeological data remained relatively constant across periods, varying between 90.4 and 98.1% of the maximum Holocene range based on all records (table 1). Sufficient data (greater than or equal to 3 records per period) were available to reconstruct former spatial distributions using range extent across all seven archaeological periods for 15 large-bodied European mammal species which had native Holocene ranges that occupied more than 5% of the study area (table 1 and figure 2; electronic supplementary material, figure S2).

**Table 1.** Body masses and geographical range estimates across seven Holocene archaeological periods for 15 large-bodied European mammal species. Number of data points available for range estimation in each archaeological period indicated for each species in parentheses. Maximum possible ranges for each time period are also shown; figures in parentheses indicate this as a percentage of the maximum possible Holocene range that can be calculated from our data based on all time periods combined.

species	body mass (kg)	geographical range (km^2^)						
		Mesolithic	Neolithic	Bronze Age	Iron Age	Roman Age	Early Medieval	Late Medieval
all data		8 726 234 (90.4%)	9 465 844 (98.1%)	9 135 686 (94.7%)	9 015 775 (93.5%)	8 943 642 (92.7%)	8 873 340 (92.0%)	9 064 829 (94.0%)
aurochs (*Bos primigenius*)	800.14	5 503 115 (232)	5 556 979 (666)	5 456 297 (329)	4 394 503 (152)	3 225 418 (113)	2 683 009 (108)	3 269 904 (99)
European bison (*Bison bonasus*)	675.88	2 296 130 (30)	5 436 931 (62)	3 194 557 (31)	2 524 580 (38)	1 732 977 (38)	1 861 469 (63)	1 934 067 (63)
Eurasian elk (*Alces alces*)	461.90	5 750 031 (113)	5 838 512 (292)	6 373 194 (199)	5 358 963 (193)	4 226 945 (148)	4 474 067 (226)	4 819 832 (201)
red deer (*Cervus elaphus*)	240.87	5 720 746 (422)	6 198 511 (1348)	6 076 671 (901)	5 988 339 (775)	5 715 711 (765)	6 004 741 (613)	5 769 680 (702)
brown bear (*Ursus arctos*)	196.29	7 368 711 (141)	7 849 141 (448)	8 974 442 (326)	8 496 701 (287)	6 084 809 (246)	5 880 987 (296)	6 824 380 (221)
wild boar (*Sus scrofa*)	84.47	7 120 297 (327)	7 477 984 (1011)	7 719 226 (594)	7 642 892 (468)	6 955 638 (385)	6 788 035 (410)	6 564 706 (433)
wolf (*Canis lupus*)	31.76	5 381 505 (105)	6 854 078 (296)	7 936 051 (184)	8 052 592 (114)	5 428 748 (84)	5 982 206 (98)	6 019 218 (96)
roe deer (*Capreolus capreolus*)	22.50	6 739 738 (302)	7 177 472 (980)	7 219 402 (555)	7 084 509 (484)	7 587 755 (407)	7 005 402 (491)	7 233 090 (575)
Eurasian lynx (*Lynx lynx*)	19.30	4 906 234 (34)	4 511 031 (105)	6 250 827 (49)	6 460 317 (44)	5 820 409 (26)	4 737 389 (39)	4 485 519 (32)
Eurasian beaver (*Castor fiber*)	19.00	7 061 248 (139)	7 595 139 (537)	8 343 810 (269)	8 457 497 (290)	6 749 772 (234)	5 865 370 (266)	5 975 811 (219)
red fox (*Vulpes vulpes*)	4.82	5 985 240 (200)	8 347 285 (577)	8 871 627 (363)	8 622 826 (296)	7 083 027 (262)	7 259 562 (259)	7 100 746 (279)
European wildcat (*Felis sylvestris*)	4.57	4 457 541 (133)	5 223 898 (320)	5 355 349 (157)	4 511 447 (84)	4 217 660 (46)	3 660 833 (36)	2 982 070 (33)
beech marten (*Martes foina*)	1.68	4 785 018 (68)	4 734 759 (185)	6 634 106 (82)	5 079 962 (46)	4 318 686 (51)	4 296 558 (67)	2 104 764 (43)
pine marten (*Martes martes*)	1.30	593 830 (4)	2 713 330 (33)	2 434 425 (26)	2 115 159 (17)	928 156 (14)	2 309 390 (10)	1 737 344 (12)
polecat (*Mustela putorius*)	0.98	2 911 800 (20)	3 607 252 (62)	3 040 819 (39)	3 222 494 (26)	3 335 084 (27)	2 465 224 (38)	1 644 229 (47)

**Figure 3.**
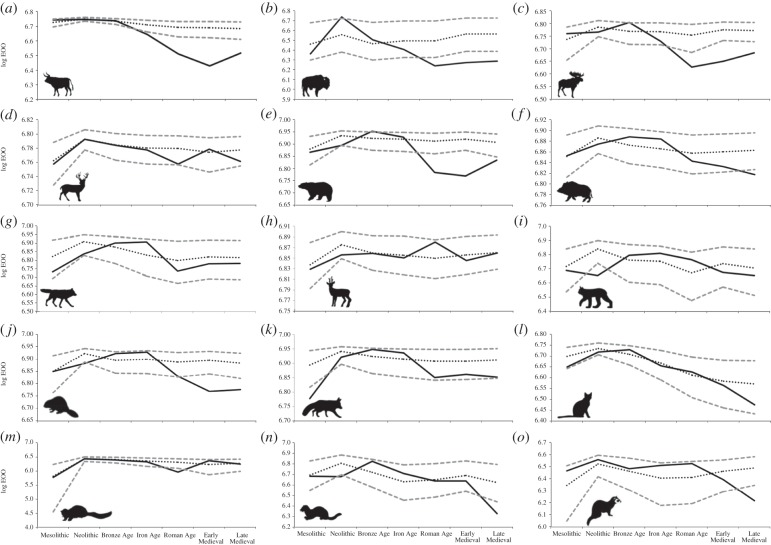
Log range extent from the Mesolithic to the Late Medieval for (*a*) aurochs, (*b*) European bison, (*c*) Eurasian elk, (*d*) red deer, (*e*) brown bear, (*f*) wild boar, (*g*) wolf, (*h*) roe deer, (*i*) Eurasian lynx, (*j*) Eurasian beaver, (*k*) red fox, (*l*) European wildcat, (*m*) beech marten, (*n*) pine marten and (*o*) polecat. Grey dashed lines denote bootstrapped 95% CIs for geographical range extents; black dotted line denotes mean range estimates from all bootstrapped samples; solid black line denotes observed ranges.

Although the raw range extent data show decreases in recorded spatial distribution for almost all of the 15 large-bodied mammal species in our dataset across the Holocene (table 1), these changes in distribution are associated with variation in the number of available zooarchaeological records between different time periods. As a conservative measure of range decline, we therefore contrasted expected range (accounting for sample size per archaeological period) against observed decline. Only eight species underwent statistically significant declines in geographical range by the Late Medieval as measured by their observed range falling below the 95% confidence intervals of the expected range controlling for sample size (figure 3). These significant declines started at different time periods across the Holocene for different species: aurochs experienced a statistically significant decline from the Iron Age; European bison (*Bison bonasus*), Eurasian elk (*Alces alces*) and brown bear (*Ursus arctos*) experienced significant declines from the Roman Age; Eurasian beaver (*Castor fiber*) experienced a significant decline from the Early Medieval; and wild boar (*Sus scrofa*), pine marten (*Martes martes*) and polecat (*Mustela putorius*) experienced significant declines in the Late Medieval. Aurochs subsequently became completely extinct across Europe during the recent historical era, while European bison became extinct in the wild. By contrast, the remaining seven species in our dataset—red deer (*Cervus elaphus*), roe deer (*Capreolus capreolus*), Eurasian lynx (*Lynx lynx*), European wildcat (*Felis silvestris*), wolf (*Canis lupus*), red fox (*Vulpes vulpes*) and beech marten (*Martes foina*)—broadly maintained their geographical ranges across Europe throughout the pre-modern Holocene. Although several of these species (e.g. lynx, wolf, wildcat) have since suffered substantial range declines from their maximum Holocene ranges [34], our data indicate that such losses probably only occurred within the last 500 years. Some ‘pseudo-declines’ early in the Holocene were generally due to a temporary absence of zooarchaeological records from outlying regions (e.g. lynx appear to decline in the Neolithic due to a lack of Neolithic–Bronze Age records in Britain, but the species reappears in Britain from the Roman Age–Early Medieval). This is unlikely to represent a genuine extinction and re-colonization event, but rather reflects the rarity of lynx in the zooarchaeological record, with only five reliable postglacial records known from Britain.

**Figure 4.**
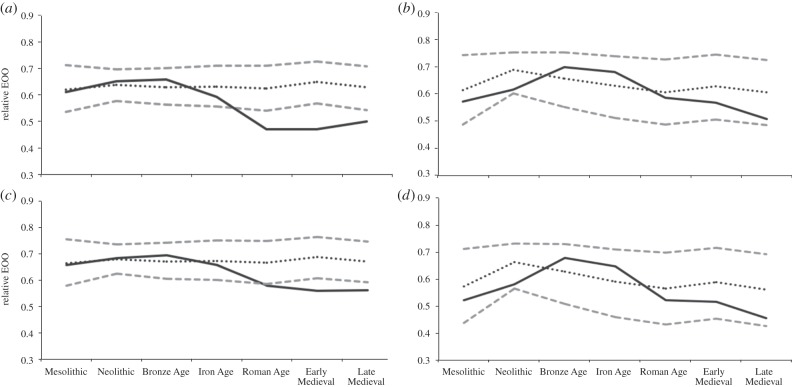
Observed mean range and expected mean range based on bootstrap models across all time periods, for (*a*) species with body mass more than 100 kg, (*b*) species with body mass less than 100 kg, (*c*) herbivores and (*d*) carnivores. All calculations are on a relative scale. Grey dashed lines denote bootstrapped 95% CIs for geographical range extents; black dotted line denotes mean range estimates from all bootstrapped samples; solid black line denotes observed ranges.

Across the European large-bodied mammalian assemblage as a whole, species with a body mass over 100 kg experienced a cumulative significant decline in geographical range by the Roman Age, whereas species under 100 kg experienced no cumulative significant range decline (figure 4*a*,*b*). Similarly, herbivores overall experienced declines by the Roman Age, whereas carnivores showed no significant decline by the Late Medieval (figure 4*c*,*d*).

## 4. Discussion

Our analysis of an extensive, approximately 11 700-year, continental-scale zooarchaeological dataset reveals that the European large mammal fauna experienced a protracted depletion of species ranges across the later part of the Holocene, with significant declines starting in some species approximately 3000 years ago and continuing into the recent historical era. This new model of continental Holocene mammalian biodiversity loss reveals that not only the globally extinct aurochs, but also over half of Europe's widely distributed large mammal fauna, underwent statistically detectable postglacial population declines before the recent historical era, with accurate identification of these losses requiring a framework of quantitative analysis that controlled for error and bias in baseline zooarchaeological data. Holocene range declines in European large mammals were non-congruent in time and space rather than representing a single easily diagnosable ‘event’, with the starting point, duration and magnitude of declines varying individually between species; some taxa became globally extinct during the Holocene, whereas others experienced limited or no significant range change over this interval. This species-specific pattern of vulnerability and resilience demonstrates that although large mammals are increasingly vulnerable to human pressures compared with other taxa [54], extinction risk represents an interaction between both extrinsic and intrinsic factors; extinction dynamics vary across species with different life histories, ecologies and geographies even if they are facing the same external threat processes [54].

Our analysis demonstrates the onset of human impacts on postglacial biodiversity long before the recent historical era, with population-level attrition of the large mammal assemblage already detectable by the Iron Age. Models of prehistoric deforestation suggested that forest loss increased across Europe from around this time (approx. 1000 BC [55]); widespread habitat loss and degradation may therefore have been a primary driver of mammal declines, at least for large herbivores such as aurochs, bison and elk, either on its own or in combination with the increased landscape access for hunting also associated with deforestation. Spatially, the earliest range losses occurred in Britain and southern Scandinavia, almost certainly reflecting the increased vulnerability of relatively small isolated mammal populations on islands [56]. Several species (such as aurochs, bison and wildcat) only colonized the southern part of Sweden, reflecting their northern latitudinal limit, which coincides closely with the distribution of deciduous woodland in Europe; when sea levels rose in the Early Holocene, these populations were isolated on ‘habitat islands’ and became similarly vulnerable to extinction. However, our results also demonstrate the lengthy time periods over which population declines took place in specific large mammal taxa at a continental scale. Total extinction of wild populations of aurochs and bison across Europe took approximately 3000 years and 2000 years, respectively, to run its course from the first evidence of spatial population decline, and millennial- or century-scale population attrition of several other species led to identifiable range reductions but continued persistence in other parts of their European ranges.

The extinction dynamics of Europe's large mammal fauna during the Holocene were characterized by temporally ‘staggered’ population extirpations, which show protracted trajectories of continental-level loss following first detectable onset of decline. This pattern provides an interesting perspective on the earlier global extinction ‘event’ of more than 90 large-bodied mammal genera during the Late Pleistocene, for which both human activity and climatic change are implicated as potential driving factors [57,58]. Radiometric and genetic data for extinct Eurasian megafaunal taxa adapted to cooler, more open environments that characterized the previous glacial period have revealed similar individual, species-specific population losses staggered across space and time, through the Late Pleistocene and in some cases into the Holocene as restricted relict populations of ‘Pleistocene survivors’ [29,59–62]. Our Holocene data demonstrate that extinctions in co-occurring large mammal faunas have therefore taken place across varying time scales for different species throughout the Late Quaternary and up to the present in Europe. The protracted trajectory of continental-level extinction seen in the Holocene record is also comparable in duration to current estimates of the extinction period for ‘naive’ regional megafaunas during the Late Pleistocene, with the human–megafauna overlap period estimated at approximately 3900 years in Australia [63] and approximately 1570 years across North and South America [64]. Our study therefore provides further evidence that continental-scale losses of large mammal populations during the Late Quaternary took place over millennial-scale time periods in response to pre-modern anthropogenic environmental pressures and technologies. However, whereas some remnant European populations of ‘Pleistocene survivors’ (e.g. *Megaloceros*) disappeared relatively early on in the Holocene [60], we demonstrate the long-term stability of Europe's Holocene large mammal fauna, with populations persisting across the continent for at least 7000 years prior to any discernible impact of prehistoric human activities on their continental-level distributions. In particular, these species resisted the impact of major anthropogenic transitions from nomadic hunter–gatherer to more sedentary agricultural lifestyles in Europe, in marked contrast to the onset of population responses shown by American and Australian Late Pleistocene mammalian megafaunas to regional human arrival.

When considered at the guild level, herbivores and larger-bodied (more than 100 kg) species in the European mammal assemblage experienced significant range declines during the Holocene, whereas carnivores and smaller-bodied species did not display significant declines before the recent historical era. These biological traits are conflated, with herbivores representing the largest-bodied mammal species in this assemblage (including brown bear, the regionally largest member of the Carnivora, but defined here as a functional herbivore for analysis), and carnivores representing the smallest-bodied species. Conversely, persecution of carnivores has intensified in recent centuries [34,36], and large carnivores are today considered to be highly vulnerable to human pressures [36–38]. It has rarely been recognized that carnivores have displayed greater historical resilience than herbivores, demonstrating an important extinction filter in our understanding of faunal vulnerability and resilience. Indeed, the loss of large herbivores before large carnivores in human-dominated landscapes may not be a phenomenon unique to Europe. Large herbivores such as short-horned buffalo (*Bubalus mephistopheles*), Père David's deer (*Elaphurus davidianus*), Sumatran rhino (*Dicerorhinus sumatrensis*), Javan rhino (*Rhinoceros sondaicus*) and Asian elephant (*Elephas maximus*) disappeared from much or all of China during the Holocene before the recent historical era [65–67], while large carnivores such as wolf, tiger (*Panthera tigris*) and leopard (*P*. *pardus*) survived across much of this large region until very recently or even into the present [68,69]. Similar patterns of differential trophic loss have also been witnessed in the large mammal faunas of other geographical regions, such as the Near East and Egypt [70–72].

Relative decreases in prey abundance have been found to result in proportionally greater declines of large carnivores [73], such that evidence for herbivore declines preceding carnivore declines during the Holocene by centuries or even millennia appears counterintuitive. However, whereas some European carnivores are specialist predators with a narrow prey range (e.g. Iberian lynx *Lynx pardinus* [74]), the domestication of pigs, sheep and cattle across Europe during the Early Holocene, and introduction of species such as fallow deer (*Dama dama*), rabbit (*Oryctolagus cuniculus*) and European hare (*Lepus europaeus*) as far north as the UK and Scandinavia [39,40,75,76], would have provided a new prey-base for non-specialist carnivores in the absence of an abundant wild prey-base [36]. Carnivores also have higher maximum natal dispersal distances than herbivores [77], making them better able to exploit landscapes across wide geographical ranges in the face of anthropogenic changes to their environment. Their reliance on secondary productivity enables them to survive in relatively degraded landscapes as long as sufficient prey or other resources are present [78,79], and this trophic ecology is also associated with broader elevational tolerance; many large carnivores can persist at both low and high elevations, and indeed are now largely restricted to mountainous areas in Europe [34]. By contrast, the European herbivore fauna shows elevational niche differentiation, comprising low-elevation species with formerly wide Holocene distributions and high-elevation specialists such as ibex (*Capra* spp.) and chamois (*Rupicapra* spp.).

Our findings emphasize the crucial importance of using large-scale, long-term environmental archives to understand the spatio-temporal dynamics of protracted, potentially complex extinction processes [58], and we encourage further use of quantitative frameworks in accurate interpretation of zooarchaeological and other Quaternary data for understanding past patterns of faunal change. Rather than simply documenting regional and global last-occurrence dates for species, palaeobiologists can instead begin to investigate new extinction paradigms, exploring the timing, duration and magnitude of losses, both for individual species and across assemblages through time, in addition to the driving forces behind these trends. Our evidence for large mammal range loss beginning as early as 3000 years ago in Europe provides a note of caution against underestimating the effects of even relatively low levels of human activity on mammalian population persistence, or the potential impact of such pressures prior to the recent historical era. These findings have important implications for reinterpreting the current population status and conservation prioritization of European large mammal species, from ungulates to mustelids to beavers and bears, which experienced previously unrecognized pre-modern population declines. However, the dynamic pattern of progressive faunal depletion and changing composition of European regional mammal assemblages observed across much of the Holocene also challenges easy identification of ‘static’ past baselines to inform current-day environmental management and restoration [80]. Conservation scientists therefore need to strengthen links in perspectives on ‘past’ and ‘present’ to understand the full scope of anthropogenic effects on biodiversity in regions with long histories of human presence.

## Supplementary Material

Text S1

## Supplementary Material

Table S1

## Supplementary Material

Figure S1

## Supplementary Material

Figure S2

## Data accessibility

The datasets supporting this article have been uploaded in map form as part of the electronic supplementary material figure S2.
