# Supplementary material for: Millennial-scale faunal record reveals differential resilience of European large mammals to human impacts across the Holocene
Source: Proc Biol Sci. 2016 Mar 30;283(1827):20152152. doi: 10.1098/rspb.2015.2152 (PMC4822451; doi:10.1098/rspb.2015.2152)
Supplement: Text S1 [file rspb20152152supp1.docx]

**Supporting Electronic Information Text S1:**

**Compiling, dating, and auditing the zooarchaeological database**

Zooarchaeological records were obtained from published and unpublished literature, grey literature, museum and institute reports, online databases and personal communication. Published identifications for species were followed, including wild/domestic status. A record was defined as the occurrence of a species within a stratigraphic layer. One or multiple bones could be present in a specific layer, but this was counted as one record so long as this material was stratigraphically related and therefore assigned the same date. Bones found within different dated stratigraphic layers from the same site were counted as separate records. Combining both databases resulted in 18,670 faunal records across 24 species, with ~85% of the data originating from the original Holocene vertebrate database (Benecke 1999) and ~15% from additional data collection.

Using other people’s data will invariably result in data that vary in precision and accuracy, and researchers have been found to generate markedly different interpretive conclusions when faced with the same data (Atici *et al*. 2013). As a result there is now increased usage of standardised ‘auditing’ techniques for dealing with directly and indirectly dated records collected from the literature and other disparate sources, in order to screen them for reliability (Stuart *et al*. 2004; Stuart 2005; Pacher and Stuart 2009; Stuart and Lister 2010). In general, we erred on the side of conservatism with regards to identification and dating of all zooarchaeological records.

In archaeology there are two main methods of dating: i) absolute dating, which provides an approximate computed date, for example through radiocarbon, thermoluminescence or potassium series dating; and ii) relative dating, which provides an order of events that can be dated in relation to each other, primarily through stratigraphy, where a higher layer may be considered younger than a lower layer that was deposited first. Due to its increased accuracy, absolute dating was preferred over relative dating, which is generally less precise than absolute dating and can sometimes be erroneous due to complicated stratigraphy that mixes up the usual ordering of layers based on age.

The following preliminary protocols were first applied to all records across the final zooarchaeological database:

- All zooarchaeological records were mapped in ArcMap 9.3 (ESRI 2008) to cross-check location information from the database and correct assignment of geographic coordinates (latitude-longitude). Where location information was incorrect, the site or location was searched for in a georeferencing facility such as iTouch (<http://itouchmap.com/latlong.html>) to obtain accurate geographic coordinate information.
- Where information for records was missing, the original reference was sought and information filled in. If crucial information on the date or location of the record could not be found, it was rejected.
- Where there had been multiple studies on a faunal assemblage or site, the most recent assessment of the data was used.
- Poorly dated records, or records with questionable dates, were rejected.
- Where multiple dates for a site or record were available, absolute dates were preferred over relative dates.
- Where several absolute dates were provided for a site, the most ancient was used for association with a record, to be conservative with regards to extinction chronologies and interpreting changes in faunal assemblages over time.
- Where sites or specimens had been re-dated, the most recent dates were used, on the assumption that theory and techniques of assessing archaeological remains have improved over time.
- All radiocarbon records where the raw radiocarbon date was cited were re-calibrated for consistency in absolute dating across the database, using the calibration curve Intcal 09 in the programme OxCal version 4.1 (Bronk Ramsey 2009) to provide an upper and lower 95% probability estimate of the date of the faunal sample (in years BC).
- Relatively dated records that spanned more than two archaeological time periods were rejected due to lack of precision.

All final records were then identified as being dated within one of five approximate categories:

| **Category** | **Dating of record** | **Number of records** |
| --- | --- | --- |
| 1. | Radiocarbon (^14^C) dated | 546 |
| 2. | Pre-assigned date from original reference related to cultural or archaeological period | 7861 |
| 3. | Assigned to a culture (e.g. Karanovo I; La Tène) | 5123 |
| 4. | Assigned to part of an archaeological period (e.g. Early Mesolithic, Middle Bronze Age; Late Iron Age) | 2106 |
| 5. | Assigned to an entire archaeological period (e.g. Mesolithic, Neolithic, Bronze Age) | 3034 |

No dating method in archaeology can return a single exact date. Even radiocarbon dating can only produce a date interval (following calibration) that is an estimate of the last few years of an animal’s life, to within around 95% accuracy. Furthermore, relative dates could not easily be used alongside direct dates without being assigned a numerical time as archaeological periods, for example, were temporally non-congruent across much of Europe due to a lag in diffusions in technology and subsistence methods (e.g. Pinhasi *et al.* 2005). Thus it was most sensible to treat all records within the database as estimated interval dates. Firstly, a dataset was compiled of all cultural periods mentioned in the zooarchaeological database, and the dates corresponding to these cultures were searched for and identified, where possible from peer-reviewed sources and also cross-referenced with more than one source. Secondly, a similar dataset was compiled for all archaeological periods mentioned in the database at the country scale for increased accuracy and to encompass the staggered dates across different parts of Europe for these periods. In order to be consistent, this was compiled mostly using the British Museum’s World Timelines website (2010) and James (2005) and for the Caucasus region from the Project ArAGATS website run by Cornell University and the Institute of Archaeology and Ethnography, NAS, Armenia (2010). All dates were converted to years BC/AD, although the majority were collected in this format already. These dates were then transferred back to all relevant records in the original database.
 Whilst not of equal length, the selected within-Holocene time periods were considered most informative in terms of reflecting specific changes in human subsistence and technology or large-scale demographic change, and thus represent changes that are likely to have had a major impact on the persistence of wild mammal populations. Non-congruity of these time periods across Europe was in general most marked during the Mesolithic and Neolithic, but more or less corresponded by the Bronze Age/Iron Age.

**References**

Atici L, Kansa SW, Lev-Tov J, Kansa EC (2013) Other people’s data: a demonstration of the imperative of publishing primary data. *J Archaeol Method Theory* 20:663-681.

Benecke, N. (ed.) (1999) *The Holocene History of the European Vertebrate Fauna: Modern Aspects of Research*(Verlag Marie Leidorf GmbH, Rahden/Westf).

British Museum (2010) *World Timelines: Europe*. Available at <http://www.worldtimelines.org.uk/world/europe>. Accessed March 15, 2013.

Bronk Ramsey C (2009) Bayesian analysis of radiocarbon dates. *Radiocarbon* 51:337-360.

Cornell University and the Institute of Archaeology and Ethnography, NAS, Armenia. 2010. *Project ArAGATS.* Available at <http://aragats.net/> Accessed March 15, 2013.

ESRI (2008) ArcMap 9.3 (ESRI, Redlands, California).

James S (2005) *The World of the Celts* (Thames and Hudson, London).

Pacher M, Stuart AJ (2009) Extinction chronology and palaeobiology of the cave bear (*Ursus spelaeus*). *Boreas* 38:189-206.

Pinhasi R, Fort J, Ammerman AJ (2005) Tracing the origin and spread of agriculture in Europe. *PLoS Biol* 3:e410.

Stuart AJ (2005) The extinction of woolly mammoth (*Mammuthus primigenius*) and straight-tusked elephant (*Palaeoloxodon antiquus*) in Europe. *Quat Int* 126:171-177.

Stuart AJ, Kosintsev P, Higham T, Lister AM (2004) Pleistocene to Holocene extinction dynamics in giant deer and woolly mammoth. *Nature* 431:684-689.

Stuart AJ, Lister AM (2010) Extinction chronology of the cave lion *Panthera spelaea*. *Quat Sci Rev* 30:2329-2340.
