# Supplementary material for: Millennial-scale faunal record reveals differential resilience of European large mammals to human impacts across the Holocene
Source: Proc Biol Sci. 2016 Mar 30;283(1827):20152152. doi: 10.1098/rspb.2015.2152 (PMC4822451; doi:10.1098/rspb.2015.2152)
Supplement: Figure S1 [file rspb20152152supp3.docx]

**Fig. S1.** Relationship between number of records and EOO for the Holocene zooarchaeological record. Each point represents one species at one time period.
