# Supplementary material for: Millennial-scale faunal record reveals differential resilience of European large mammals to human impacts across the Holocene
Source: Proc Biol Sci. 2016 Mar 30;283(1827):20152152. doi: 10.1098/rspb.2015.2152 (PMC4822451; doi:10.1098/rspb.2015.2152)

Fig. S2. Reconstructed mammal species range maps for each Holocene archaeological period.

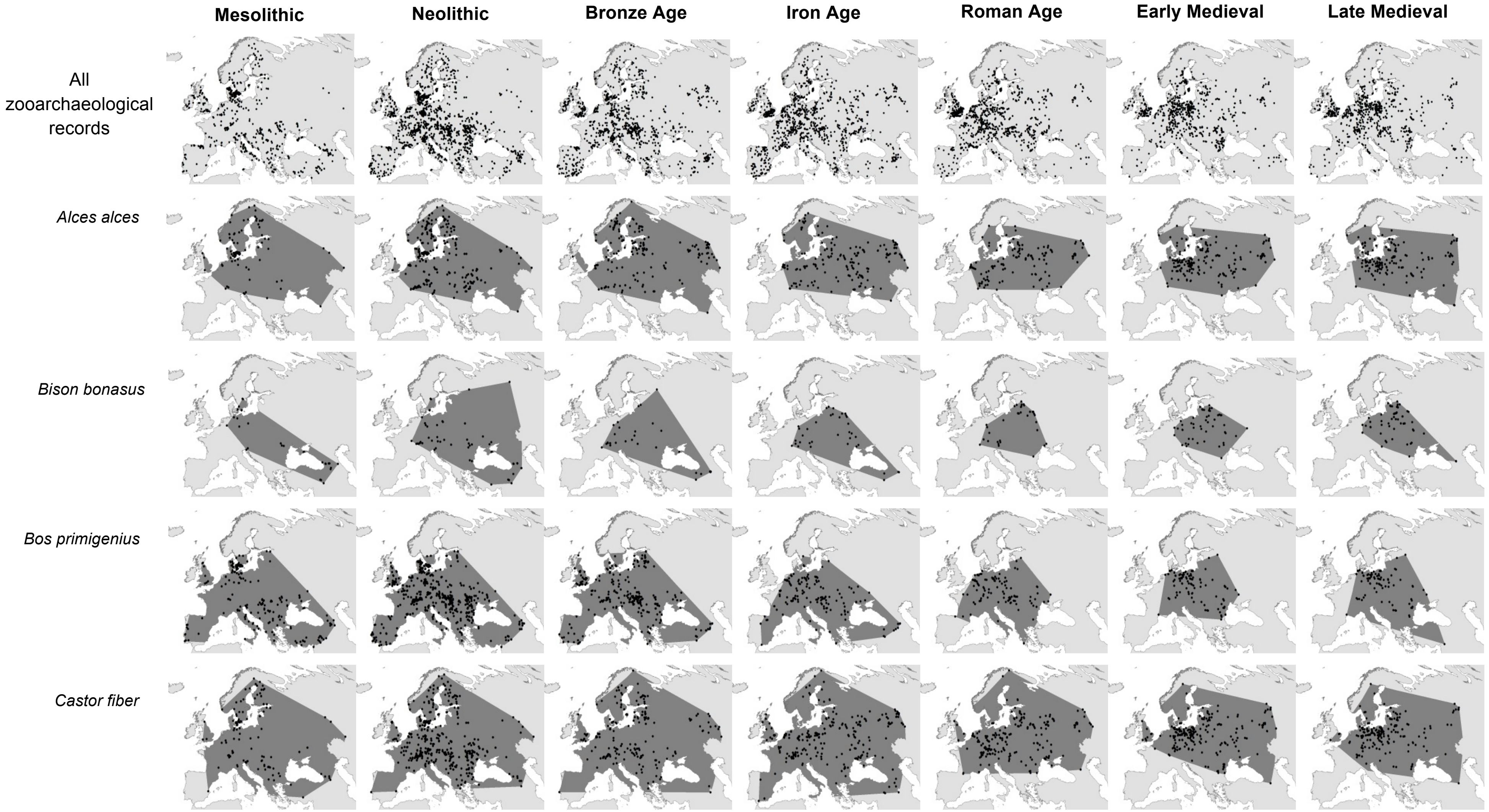

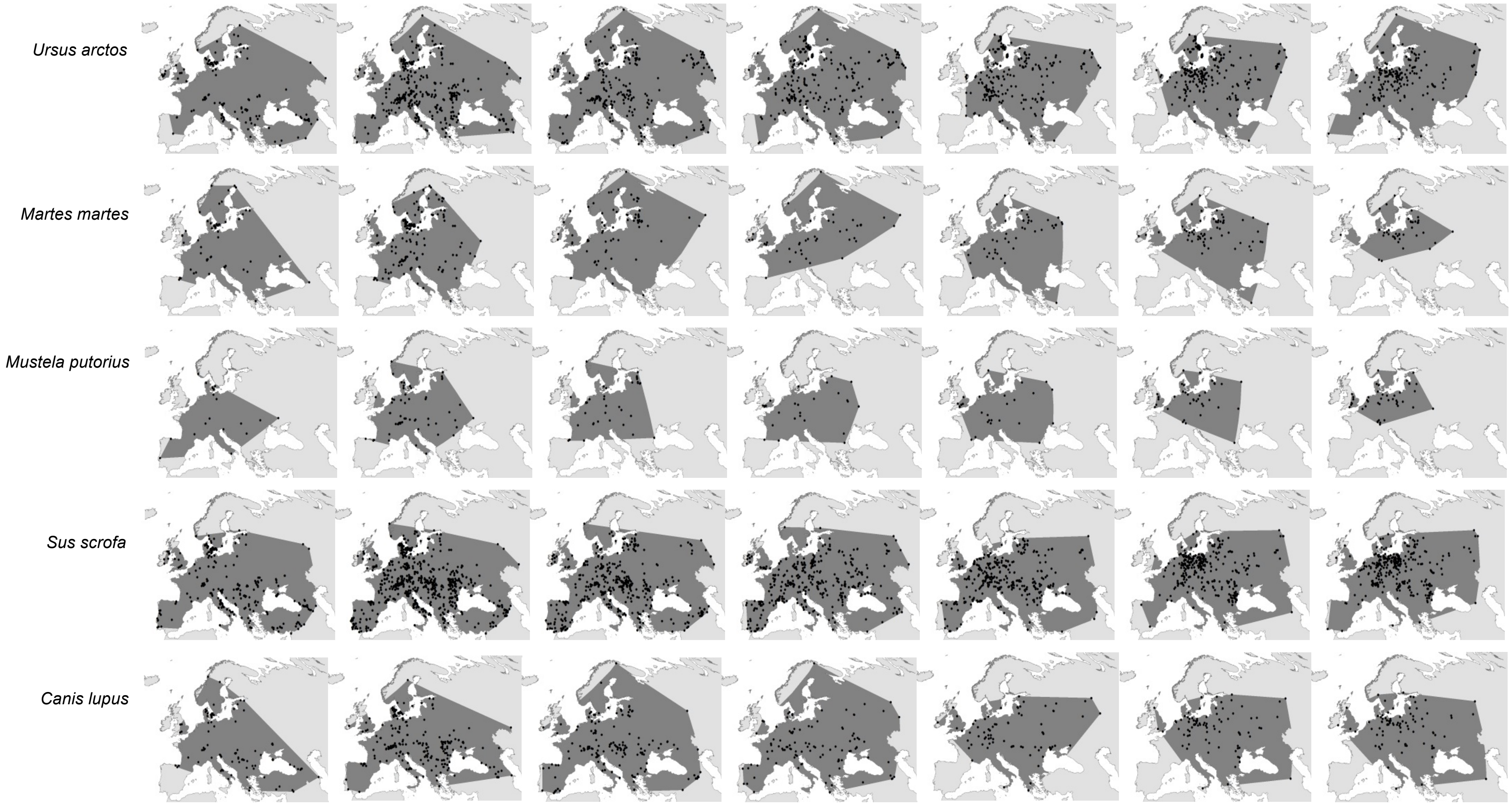

Ursus arctos
Martes martes
Mustela putorius
Sus scrofa
Canis lupus

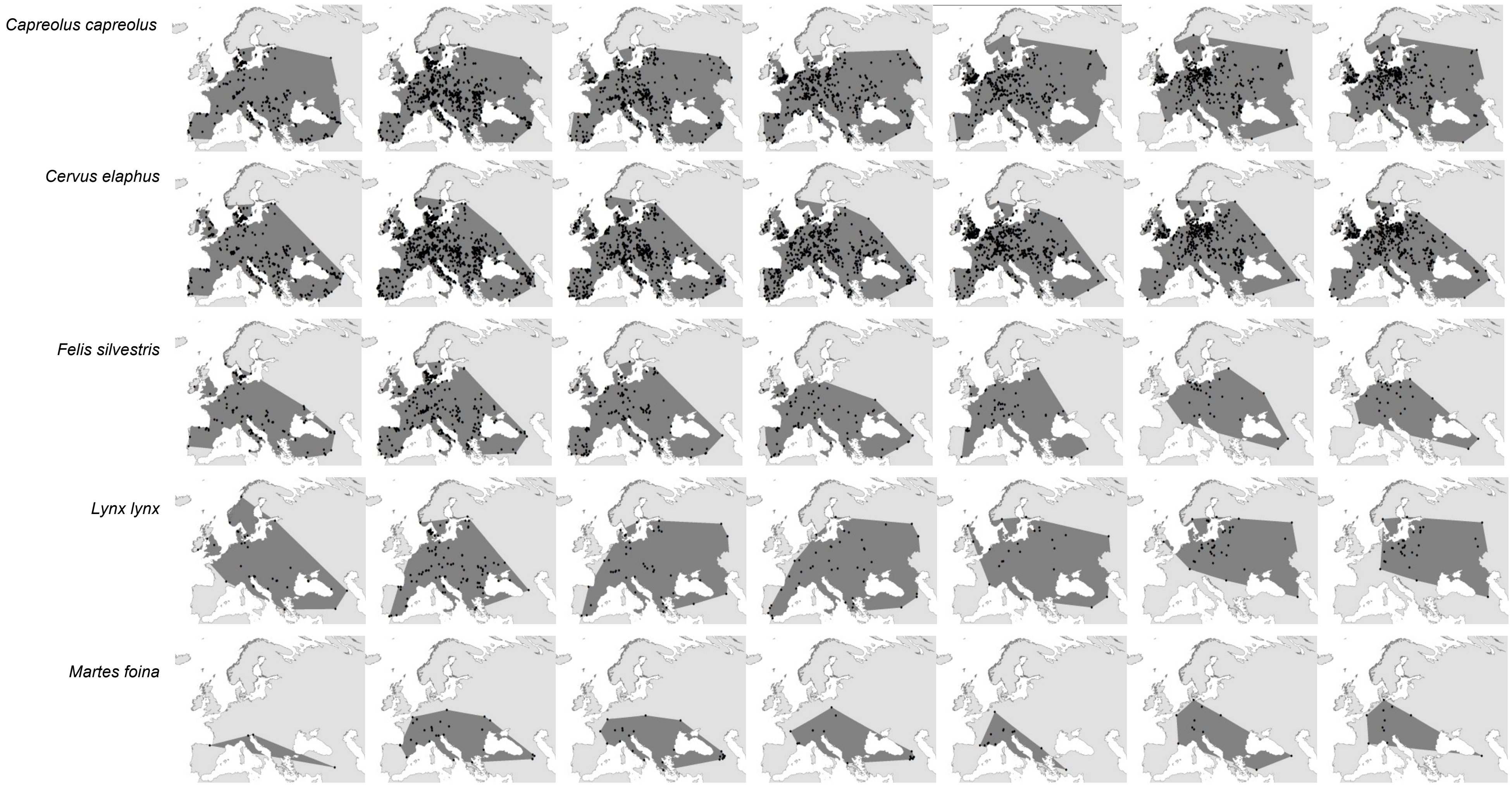

Capreolus capreolus
Cervus elaphus
Felis silvestris
Lynx lynx
Martes foina

Vulpes vulpes

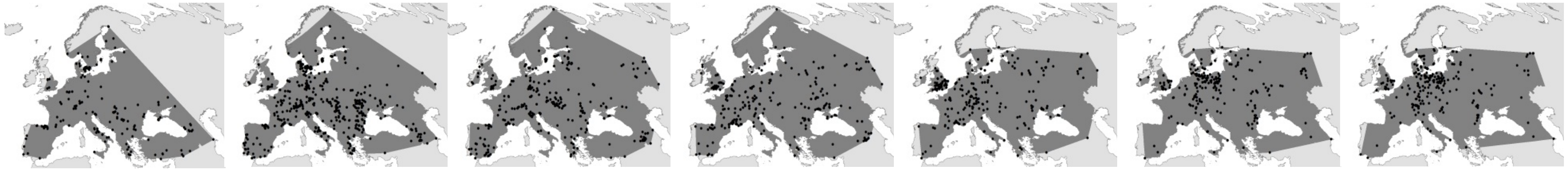

Supplement: Figure S2 [file rspb20152152supp4.pdf]
